# Association between triglyceride–glucose index, inflammation, and mitral annular calcification

**DOI:** 10.1097/MD.0000000000046313

**Published:** 2026-05-12

**Authors:** Mehmet Mustu, Damla Yalcinkaya Oner, Kadir Karacali, Hakan Suygun, Fatma Ozpamuk Karadeniz

**Affiliations:** a Department of Cardiology, Karamanoglu Mehmetbey University, Karaman Research and Training Hospital, Karaman, Turkey; b Department of Cardiology, Karaman Training and Research Hospital, Karaman, Turkey; c Department of Cardiology, Golbasi Sehit Ahmet Ozsoy State Hospital, Konya, Turkey.

**Keywords:** inflammation, inflammatory markers, insulin resistance, mitral annular calcification, triglyceride–glucose index

## Abstract

Mitral annular calcification (MAC), traditionally considered an age-related degenerative process, is now recognized as an active condition sharing pathophysiological features with atherosclerosis. While metabolic dysfunction and chronic inflammation are associated with cardiovascular calcification, their combined role in MAC remains unclear. This observational study enrolled 259 patients (130 MAC+, 129 MAC−) undergoing echocardiography. We calculated the triglyceride–glucose (TyG) index as Ln (fasting triglycerides [mg/dL] × fasting plasma glucose [mg/dL]/2) and derived inflammatory markers (neutrophil-to-lymphocyte [N/L] ratio, lymphocyte-to-monocyte [L/M] ratio, systemic immune-inflammation index) from complete blood counts. Multivariate logistic regression and receiver operating characteristic analyses were used to assess the predictors of MAC. MAC+ patients showed higher TyG index (9.02 ± 0.58 vs 8.76 ± 0.52, *P* < .001), N/L ratio (*P* < .001), and lower L/M ratio (*P* = .001). TyG index > 8.76 predicted MAC with 64.6% sensitivity (area under the curve = 0.621, *P* = .001), while N/L ratio > 2.08 showed 60.8% sensitivity (area under the curve = 0.615, *P* = .001). In multivariate analysis, age (odds ratio [OR], 1.09, *P* < .001), TyG index (OR, 2.76, *P* = .001), and L/M ratio (OR, 0.79, *P* = .008) independently predicted MAC. MAC is associated with insulin resistance (reflected by TyG index) and systemic inflammation (elevated N/L ratio, reduced L/M ratio). These findings position MAC as a multifactorial process linked to metabolic and inflammatory pathways. The TyG index, as a simple and cost-effective marker, could potentially be used alongside inflammatory markers in risk stratification for MAC. Future studies should investigate if modulating these factors can alter MAC progression.

## 1. Introduction

Mitral annular calcification (MAC) is a chronic degeneration of the fibrous support structure of the mitral valve.^[1,2]^ The prevalence of MAC varies from 5% to 42%.^[3]^ In the past, it was considered as a passive degenerative consequence of aging. Today, it is described as an active process that shows histological similarities with atherosclerotic cardiovascular disease (CVD).^[4]^ It was previously demonstrated that MAC is closely associated with systemic atherosclerotic processes, shares common risk factors with atherosclerotic diseases, and may represent a distinct clinical manifestation of atherosclerosis.^[5]^

Metabolic syndrome, which includes obesity, insulin resistance (IR), hypertension (HTN), and hyperlipidemia (HL), is increasing as a result of a sedentary lifestyle and poor dietary habits.^[6]^ IR, a central component of metabolic syndrome, drives atherosclerotic progression by disrupting glucose and lipid metabolism, leading to hyperinsulinemia, hypertriglyceridemia, and reduced high-density lipoprotein (HDL) levels.^[7]^ The triglyceride–glucose (TyG) index, calculated as Ln (fasting triglycerides [TG; mg/dL] × fasting plasma glucose [FPG; mg/dL]/2), is a simple, cost-effective marker of IR and a predictor of atherosclerotic CVD risk.^[8,9]^

Beyond metabolic dysfunction, emerging evidence also implicates systemic inflammation in MAC pathogenesis. Inflammatory markers such as the neutrophil-to-lymphocyte (N/L) ratio and systemic immune-inflammation index (SII) reflect chronic low-grade inflammation and are associated with cardiovascular calcification and atherosclerosis.^[10,11]^ While metabolic dysfunction and chronic inflammation are associated with cardiovascular calcification, their combined role in MAC remains unclear. In this study, we aimed to investigate the relationship between MAC and the TyG index, while also evaluating the potential roles of SII and hemogram-related other inflammatory indices in MAC development. We hypothesized that both IR and systemic inflammation would be independently associated with the presence of MAC. We wanted to determine if these biomarkers could provide incremental value in identifying patients at risk for MAC, thereby contributing to a better understanding of its multifactorial pathophysiology.

## 2. Materials and methods

### 2.1. Study population

This observational screening study consecutively enrolled patients presenting to the cardiology outpatient clinic between January and July 2022 who were diagnosed with MAC on transthoracic echocardiography. From an initial pool of 295 eligible participants, we excluded 36 MAC+ patients due to incomplete laboratory or echocardiographic records, as these gaps precluded the calculation of our primary study variables. The remaining 259 patients were included in the study and divided into 2 groups: 130 patients in the MAC+ group and 129 patients in the MAC− group.

The exclusion criteria included severe valvular heart disease, rheumatic fever history, prosthetic valves, decompensated heart failure, active malignancy, renal/hepatic dysfunction, chronic inflammatory conditions, active infections, and hematologic disorders. Patients with chronic kidney disease (CKD), or chronic inflammatory conditions were excluded to minimize the potential for confounding, as these conditions are known to influence systemic inflammation, metabolic parameters, and vascular calcification.

Our study complied with the Declaration of Helsinki and was approved by the local ethical committee of the Karamanoglu Mehmetbey University, Karaman, Turkey. Patients or the public were not involved in the design, conduct, reporting or dissemination plans of our study.

### 2.2. Data collection and analysis

Information on demographic characteristics and previously diagnosed diseases was retrieved from the hospital’s electronic medical records system. HTN was defined as a systolic blood pressure ≥ 140 mm Hg and diastolic blood pressure ≥ 90 mm Hg and/or a history of anti-HTN treatment at enrollment. Diabetes mellitus (DM) was defined as a FPG > 126 mg/dL, hemoglobin A1c > 6.5%, or history of antidiabetic medications. HL was defined as a total cholesterol level > 240 mg/dL. Coronary artery disease (CAD) was diagnosed by a history of myocardial infarction, revascularization or an angiographic stenosis > 50% at least 1 major coronary artery.

### 2.3. Laboratory examination and TyG index calculation

Blood samples taken on the day of outpatient clinic admission were recorded from the database. Routine blood tests included; complete blood count, FPG (mg/dL), serum biochemical tests, kidney and liver functions, C-reactive protein (mg/dL), HDL (mg/dL), low-density lipoprotein (LDL, mg/dL), TG (mg/dL), and total cholesterol (mg/dL). N/L, platelet/lymphocyte and lymphocyte/monocyte (L/M) ratios were calculated from complete blood count. An automated hematology analyzer (Mindray BC-6000) was used to measure hematological indices. In addition, creatinine, serum electrolytes, serum cholesterol levels and detailed liver function tests were measured with the Beckman Coulter AU5800 modular analyzer. TyG index was calculated with the formula “Ln (fasting TG [mg/dL] × FPG [mg/dL]/2).” The SII was calculated by dividing the platelet count by the N/L ratio.

### 2.4. Transthoracic echocardiography examination

The echocardiographic assessment was performed using a VIVID 7 Dimension Cardiovascular Ultrasound System (Vingmed-General Electric, Horten, Norway) with a 3.5 MHz transducer. Echocardiography was performed on each patient in the left decubitus position. Left atrial diameter, left ventricular (LV) end-systolic diameter, LV end-diastolic diameter, posterior wall thickness, and interventricular septum thickness were calculated and recorded in millimeters. LV ejection fraction was calculated by the modified Simpson method. MAC was identified as a dense echogenic structure located at the atrioventricular groove adjacent to the posterior or anterior mitral leaflet on apical 2- or 4-chamber views.^[12]^

### 2.5. Statistical analysis

All analyses were carried out with SPSS version 25.0 (IBM^®^ Corp., Armonk). Kolmogorov–Smirnov and Shapiro–Wilk tests were used for normality analysis. Age and TyG index with normal distribution were shown as the mean (standard deviation) and variables with abnormal distribution were shown as the median (interquartile range). Numeric variables with normal distribution were compared using independent samples *t*-test and without normal distribution were analyzed with the Mann–Whitney *U* test between the groups. Categorical variables were compared using the chi-square Fischer-exact test. All variables associated with MAC were analyzed using univariate and multivariate logistic regression analyses. Variables detected to show a significant relation in bivariate analysis were checked for collinearity. Any detection of a strong correlation leads to the exclusion of one of the 2 variables, from the model (i.e., “TyG index” had a correlation coefficient of 0.795 with “TG” and 0.608 with “glucose.” “TyG index” was selected for the model). Statistical significance was determined as *P* < .05. Receiver operating characteristic (ROC) analysis for predicting the MAC was conducted to determine the best cutoff value using the Youden index (sensitivity + specificity − 1) of TyG index, N/L ratio, and SII.

## 3. Results

After excluding 36 MAC+ patients due to incomplete data, the study included a total of 259 patients divided into 2 groups; MAC+ group, N = 130 and MAC− group, N = 129. Demographic features and laboratory findings were demonstrated in Table 1. The patients were older in the MAC+ group compared to MAC− group (mean age; 72 [64.7–76] vs 61 [53.5–67.5], respectively, *P* < .001). Smoking ratio were also significantly higher in the MAC+ group (*P* = .012). The female/male ratio was similar between groups (*P* > .05). The prevalence of DM, HL, CAD and CKD was significantly higher in the MAC+ group (*P* = .003, *P* = .020, *P* < .001, and *P* = .049, respectively). The prevalence of HTN was similar between 2 groups (*P > *.05). FPG, triglyceride and LDL levels were significantly higher in the MAC+ group, while HDL levels were significantly lower (*P* = .015, *P* = .021, *P* < .001, and *P* < .001, respectively).

**Table 1:** Baseline characteristics of the study groups.

Variables	All group (n = 259)	MAC (+; n = 130)	MAC (−; n = 129)	*P*-value
Age	66 (58–74)	72 (64.7–76)	61 (53.5–67.5)	**<.001** ‡
Female, n (%)	165 (63.7)	87 (66.9)	78 (60.5)	.280*
Diabetes, n (%)	146 (56.4)	85 (65.4)	61 (47.3)	**.003** *
Hypertension, n (%)	141 (54.4)	76 (58.5)	65 (50.4)	.192*
HL, n (%)	113 (43.6)	66 (50.8)	47 (36.4)	**.020** *
CAD, n (%)	130 (50.2)	76 (58.5)	54 (41.9)	**.001** *
CKD, n (%)	53 (20.5)	33 (25.4)	20 (15.5)	**.049** *
Smoker, n (%)	35 (13.5)	19 (14.6)	16 (12.4)	.603*
FPG (mg/dL)	92 (81–133)	94 (82–137)	90 (79–103)	**.015** ‡
Creatinine (mg/dL)	0.84 (0.71–1.08)	0.85 (0.75–1.10)	0.83 (0.65–1.10)	**.031** ‡
Total cholesterol (mg/dL)	185.4 ± 45.3	188.4 ± 45.6	182.4 ± 44.9	.289†
Triglyceride (mg/dL)	141 (103–184)	151 (106–197)	130 (103–171)	**.021** ‡
LDL (mg/dL)	78 (52–122)	104 (75–140)	80 (48–117)	**<.001** ‡
HDL (mg/dL)	53 (41–84)	47 (37–55)	57 (45–94)	**<.001** ‡
WBC count (10^9^/L)	7.82 (6.56–9.80)	7.95 (6.49–10.0)	7.7 (6.78–9.69)	.622‡
Neutrophil count (10^9^/L)	4.82 (3.72–6.19)	5.22 (3.95–6.53)	4.54 (3.55–5.83)	.081‡
Lymphocyte count (10^9^/L)	2.25 (1.74–2.70)	2.11 (1.62–2.65)	2.39 (2.01–2.77)	**.002** ‡
Monocyte count (10^9^/L)	0.51 (0.41–0.64)	0.54 (0.42–0.67)	0.47 (0.40–0.59)	**.026** ‡
Hemoglobin (g/dL)	13.1 (11.8–14.4)	12.5 (11.2–13.9)	13.6 (12.5–14.7)	**<.001** ‡
Platelet count (10^9^/L)	252 (212–319)	252 (207–302)	248 (216–327)	.261‡
TyG index	8.89 ± 0.56	9.02 ± 0.58	8.76 ± 0.52	**<.001** †
N/L ratio	2.2 (1.6–2.9)	2.4 (1.7–3.4)	2.1 (1.6–2.5)	**.001** ‡
P/L ratio	115.6 (88.6–150.6)	118.7 (92.1–155.9)	108.5 (82.6–149.1)	.150‡
L/M ratio	4.4 (3.3–5.7)	3.7 (2.9–5.3)	4.9 (3.8–6.2)	**.001** ‡
SII	564.5 (377.1–783)	580.7 (405.5–828.3)	536.8 (354.2–763.8)	.083‡
LVEF	60 (55–60)	60 (55–60)	60 (55–60)	.093‡

Bold values indicate statistically significant differences (*P* < .05).

CAD = coronary artery disease, CKD = chronic kidney disease, FPG = fasting plasma glucose, HDL = high-density lipoprotein, HL = hyperlipidemia, L/M = lymphocyte/monocyte, LDL = low-density lipoprotein, LVEF = left ventricular ejection fraction, MAC = mitral annular calcification, N/L = neutrophil/lymphocyte, P/L = platelet/lymphocyte, SII = systemic inflammatory index, TyG = triglycerides glucose, WBC = white blood cell.

* Chi-square Fischer-exact test.

† Independent samples *t*-test.

‡ Mann–Whitney *U* test.

**Figure 1.**
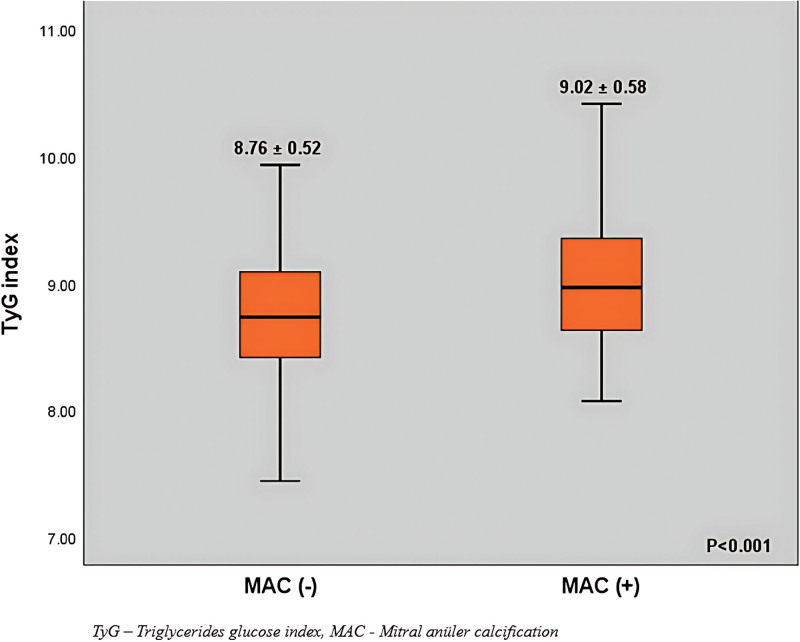
Box-plot graphs of triglyceride-glucose indexes according to the groups. MAC = mitral annular calcification, TyG = triglyceride-glucose.

Lymphocyte count was significantly lower while monocyte count was higher in the MAC+ group (*P* = .002, and *P* = .026, respectively). Thus L/M ratio was lower in the MAC+ group compared to MAC− group (*P* = .001). TyG index was 8.89 ± 0.56 in the whole population and was significantly higher in the MAC+ group compared to MAC− group (9.02 ± 0.58 vs 8.76 ± 0.52, *P* < .001, respectively; Fig. 1). N/L ratio was significantly higher in the MAC+ group (*P* < .001).

**Figure 2.**
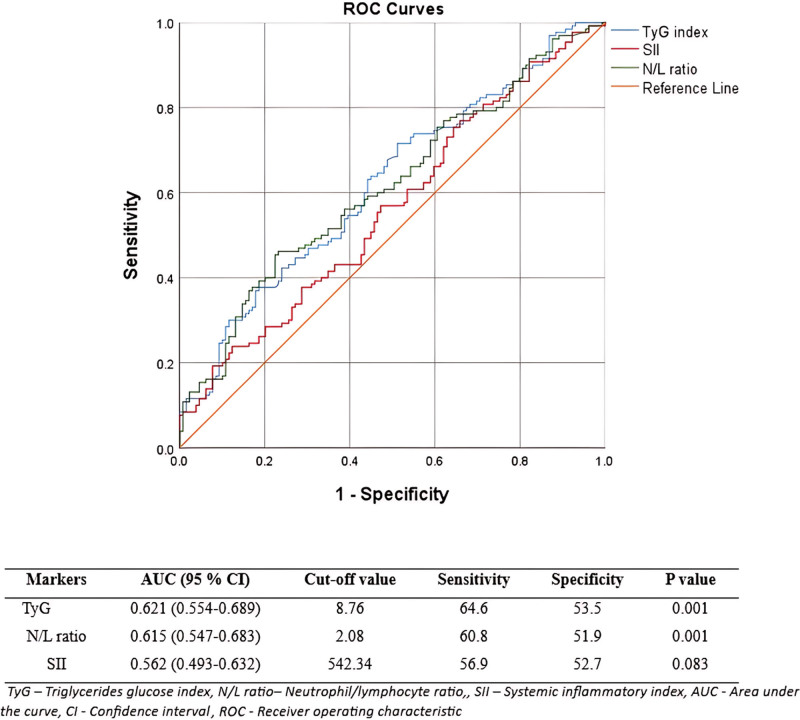
Receiver operating characteristics curve analysis for determining the predictive value of the triglyceride-glucose index, neutrophil-to-lymphocyte ratio and systemic immune-inflammation index for the presence of mitral annular calcification. AUC = area under the curve, CI = confidence interval, N/L = neutrophil-to-lymphocyte, OR = odds ratio, ROC = receiver operating characteristic, SII = systemic immune-inflammation index, TyG = triglyceride-glucose.

Figure 2 indicated the ROC curves of TyG index, N/L ratio and SII levels to predict MAC. TyG levels above 8.76 predicted MAC with a sensitivity of 64.6% and specificity of 53.5% (area under the curve [AUC] = 0.621 [95% CI: 0.554–0.689], *P* = .001). N/L ratio above 2.08 also predicted MAC with a sensitivity of 60.8% and specificity of 51.9% (AUC = 0.615 [95% CI: 0.547–0.683], *P* = .001). Although these AUC values indicate moderate predictive accuracy, the identification of these simple, routinely available biomarkers highlights their potential clinical utility as supportive tools in the comprehensive assessment of cardiovascular risk, rather than as stand-alone diagnostic tests for MAC. SII showed a tendency toward predicting MAC; however, the association was not statistically significant (*P* = .083).

Results of univariate and multivariate analysis were shown in Table 2. Statistically significant predictors of MAC were age (odds ratio [OR], 1.1 [1.07–1.14]; *P < *.001), DM (OR, 2.11 [1.28–3.47]; *P* = .004), HL (OR, 1.8 [1.09–2.96]; *P* = .02), CKD (OR, 1.85 [0.99–3.44]; *P* = .051), CAD (OR, 1.96 [1.19–3.20]; *P* = .008), triglyceride (OR, 1.01 [1.00–1.09]; *P* = .011), FPG (OR, 1.01 [1.00–1.02]; *P* = .003), TyG index (OR, 2.47 [1.53–3.99]; *P* < .001), L/M ratio (OR, 0.75 [0.65–0.86]; *P < *.001), and N/L ratio (OR, 1.40 [1.14–1.73]; *P = *.002). In multivariable analysis, age (OR, 1.09 [1.06–1.13]; *P < *.001), L/M ratio (OR, 0.79 [0.68–0.94]; *P = *.008), and TyG index (OR, 2.76 [1.52–5.01]; *P = *.001), were the independent predictors of MAC.

**Table 2:** The univariate and multivariate analysis of parameters possibly associated with mitral annular calcification.

Variables	Univariable analysis		Multivariable analysis	
	OR (95% CI)	*P*-value	OR (CI)	*P*-value
Age	1.10 (1.07–1.14)	**<.001**	1.09 (1.06–1.13)	**<.001**
Diabetes	2.11 (1.28–3.47)	**.004**	1.69 (0.92–3.12)	.091
Hyperlipidemia	1.80 (1.09–2.96)	**.020**	1.74 (0.94–3.21)	.076
CKD	1.85 (0.99–3.44)	**.051**	1.38 (0.65–2.92)	.399
CAD	1.96 (1.19–3.20)	**.008**	1.39 (0.76–2.51)	.284
Triglyceride	1.01 (1.00–1.09)	**.011**		
Glucose	1.01 (1.00–1.02)	**.003**		
TyG index	2.47 (1.53–3.99)	**<.001**	2.76 (1.52–5.01)	**.001**
N/L ratio	1.40 (1.14–1.73)	**.002**	1.21 (0.96–1.52)	.111
L/M ratio	0.75 (0.65–0.86)	**<.001**	0.79 (0.68–0.94)	**.008**

Bold values indicate statistically significant differences (*P* < .05).

CAD = coronary artery disease, CI = confidence interval, CKD = chronic kidney disease, L/M = lymphocyte/monocyte, N/L = neutrophil/lymphocyte, OR = odds ratio, TyG = triglycerides glucose.

## 4. Discussion

In this study, we investigated the relationship between MAC and TyG index, a marker of IR, while also exploring associations with systemic inflammatory markers. We found a significant association between them, suggesting that IR, as reflected by the TyG index, may play a potential role in MAC pathogenesis. Additionally, elevated N/L and decreased L/M ratios in MAC + patients suggest that chronic low-grade inflammation may further contribute to valvular calcification. Our results are consistent with the idea that MAC is an active process similar to atherosclerosis, rather than a passive degenerative change.

MAC is characterized by calcium deposition that more commonly affects the posterior side of the mitral annulus than the anterior annulus.^[13]^ While MAC is a local calcification of the fibrous skeleton in the mitral annulus, it also results from systemic diseases such as disturbances in lipid and calcium metabolism, CKD, and inflammation.^[14]^ Histological examinations have linked MAC to atherosclerosis, demonstrating foam cell infiltration and lipid deposition similar to that seen in CAD.^[15]^ Although the prevalence of MAC increases with aging, its progression is driven by risk factors that overlap with those of atherosclerosis.^[16]^ Studies have demonstrated that MAC is closely associated with systemic atherosclerotic processes, shares common risk factors with atherosclerotic diseases, and may represent a distinct clinical manifestation of atherosclerosis.^[17]^

The TyG index is calculated using fasting levels of TG and glucose.^[8]^ This index provides a simpler and alternative way to assess IR compared to conventional methods like the homeostasis model assessment for IR.^[18]^ Studies have demonstrated that the TyG index possesses high sensitivity and specificity in indicating IR and metabolic syndrome. Wan et al found the TyG index to be a slightly better marker than homeostasis model assessment in predicting metabolic syndrome.^[19]^ Guerrero-Romero et al further demonstrated that TyG index had high sensitivity and specificity to recognize IR, suggesting that it could be useful for the identification of patients with decreased insulin sensitivity.^[20]^ Besides from IR, this contemporary index has been shown to independently predict increased risk of atherosclerotic CVD even in individuals without established disease. A high TyG index was found to have a significant relationship with the incidence of CAD, myocardial infarction and CVD in the general population.^[21]^

Our hypothesis that the TyG index could predict MAC was based on its established role in atherosclerosis risk assessment. Consistent with previous research, our findings indicate that patients with MAC exhibited higher rates of CAD and classical risk factors, including DM and HL. The significant TyG-MAC relationship we observed suggests this index may help evaluate MAC risk, similar to its known associations with other atherosclerotic diseases. The fact that higher TyG values, along with the expected metabolic disorders (high FPG, hypertriglyceridemia, higher levels of LDL, and total cholesterol) and smoking frequency, supports that MAC is a systemic disease and it overlaps with metabolic dysfunction. Insulin is one of the essential hormones involved in glucose and lipid metabolism. IR, known as decreased insulin sensitivity, is known as the distinguishing feature of type 2 DM before the definitive diagnosis.^[22]^ In the chronic process, IR is associated with metabolic disorders that cause poor cardiovascular outcomes such as hyperglycemia, HL, and HTN. Therefore, it is important to develop inexpensive, available, reliable, and readily available screening tools for detecting IR (and thus predicting cardiovascular risks).^[23,24]^

In our study, we found that patients with MAC+ exhibited higher levels of IR, as indicated by the higher TyG index. This finding also supports the idea that MAC is not only a localized condition but also linked to broader metabolic dysfunctions. The presence of IR in MAC+ patients may contribute to adverse cardiovascular outcomes, as IR is associated with complications like high blood sugar, abnormal lipid levels, and HTN. Identifying patients with MAC for further evaluation of IR could therefore be crucial in managing their cardiovascular risk. The ROC analysis revealed that TyG index levels above 8.76 predicted MAC with a sensitivity of 64.6% and specificity of 53.5%. This suggests that the TyG index has a moderate ability to predict individuals with and without MAC in our study population.

Our findings also highlight that MAC+ patients had significantly higher chronic systemic inflammation, as evidenced by elevated N/L and decreased L/M ratios. These results align with the evidence that chronic low-grade inflammation contributes to valvular calcification through a similar mechanism as in the systemic atherosclerotic process and shared inflammatory pathways.^[25,26]^ The N/L ratio, indicative of systemic inflammation, has been associated with CVD and may reflect the inflammatory component in MAC pathogenesis.^[10]^ In our study, N/L ratio above 2.08 also predicted MAC with a sensitivity of 60.8% and specificity of 51.9%. We also found a significantly lower L/M ratio in MAC+ patients (*P* = .001), suggesting an imbalance between pro-inflammatory monocytes and anti-inflammatory lymphocytes in valvular calcification. Similar to its role in demonstrating coronary atherosclerosis severity, it may also play a significant role in detecting valvular calcification.^[27]^ The SII, which combines platelet count and N/L ratio, has been proposed as a comprehensive marker of systemic inflammation and has shown value in prognostic assessment and risk stratification in various cardiovascular conditions.^[11]^ While the *P*-value for the SII in the ROC analysis (*P* = .083) did not reach statistical significance, its proximity to the .05 threshold suggests a potential trend. This indicates that while the SII alone may not be a strong predictor of MAC in our study, further investigation with larger sample sizes may reveal a more definitive relationship.

Our study has several limitations. First, its cross-sectional design prevents us from establishing a causal relationship between the TyG index and MAC development. Second, the relatively small sample size, which was not based on a pre-specified power calculation, and single-center nature may limit the generalizability of our findings. Third, although we adjusted for major confounders, residual confounding factors (e.g., dietary habits, physical activity levels, and genetic predisposition) could influence the results. Fourth, the TyG index, while practical, remains an indirect measure of IR; comparative studies with gold standard methods would strengthen its validity. In addition, we investigated the relationship between inflammatory markers and MAC; however, we did not study C-reactive protein which is the gold standard to demonstrate systemic inflammation. Due to the retrospective nature of the study and the lack of data on C-reactive protein levels, we were unable to include CRP in our analysis. Finally, our exclusion criteria (e.g., renal dysfunction, chronic inflammation) may have introduced selection bias, as these conditions are known to influence both MAC and metabolic parameters. Prospective, multicenter studies with long-term follow-up are needed to validate our observations.

## 5. Conclusion

In this cross-sectional study, we observed a significant association between MAC and both IR and systemic inflammation. These findings align with the idea that MAC is an active pathological process, driven by metabolic dysfunction and chronic low-grade inflammation, rather than a degenerative consequence of aging. The TyG index, as a simple and accessible marker of IR, may serve as a potential adjunctive clinical tool for identifying individuals at risk for MAC, particularly in the context of metabolic syndrome. Furthermore, the association between inflammatory markers and MAC suggests that immune dysregulation may also be involved in valvular calcification. Future studies should look into whether early treatments that focus on improving metabolism, like controlling blood sugar, managing lipids, and making lifestyle changes, could help slow down the progression of MAC and reduce related cardiovascular risks.

## Author contributions

**Conceptualization**: Mehmet Mustu.

**Data curation**: Mehmet Mustu.

**Formal analysis**: Kadir Karacali.

**Methodology**: Mehmet Mustu, Fatma Ozpamuk Karadeniz.

**Supervision**: Hakan Suygun, Fatma Ozpamuk Karadeniz.

**Writing** – **original draft**: Mehmet Mustu, Damla Yalcinkaya Oner.

**Writing** – **review & editing**: Mehmet Mustu, Damla Yalcinkaya Oner, Kadir Karacali.
